# Comparison of cerebral blood flow acquired by simultaneous [^15^O]water positron emission tomography and arterial spin labeling magnetic resonance imaging

**DOI:** 10.1038/jcbfm.2014.92

**Published:** 2014-05-21

**Authors:** Ke Zhang, Hans Herzog, Jörg Mauler, Christian Filss, Thomas W Okell, Elena Rota Kops, Lutz Tellmann, Thomas Fischer, Burkhard Brocke, Walter Sturm, Heinz H Coenen, N Jon Shah

**Affiliations:** 1 Institute of Neuroscience and Medicine—4: Medical Imaging Physics, Forschungszentrum Jülich, Jülich, Germany; 2 FMRIB Centre, Nuffield Department of Clinical Neurosciences, University of Oxford, Oxford, UK; 3 Department of Psychology, Dresden University of Technology, Dresden, Germany; 4 Department of Neurology, University of Aachen, Aachen, Germany; 5 Institute of Neuroscience and Medicine—5: Nuclear Chemistry, Forschungszentrum Jülich, Jülich, Germany; 6 JARA Brain—Translational Brain Medicine, Germany

**Keywords:** arterial spin labeling, cerebral blood flow, magnetic resonance imaging, MR/PET, positron emission tomography, [^15^O]water PET

## Abstract

Until recently, no direct comparison between [^15^O]water positron emission tomography (PET) and arterial spin labeling (ASL) for measuring cerebral blood flow (CBF) was possible. With the introduction of integrated, hybrid magnetic resonance (MR)-PET scanners, such a comparison becomes feasible. This study presents results of CBF measurements recorded simultaneously with [^15^O]water and ASL. A 3T MR-BrainPET scanner was used for the simultaneous acquisition of pseudo-continuous ASL (pCASL) magnetic resonance imaging (MRI) and [^15^O]water PET. Quantitative CBF values were compared in 10 young healthy male volunteers at baseline conditions. A statistically significant (*P*<0.05) correlation was observed between the two modalities; the whole-brain CBF values determined with PET and pCASL were 43.3±6.1 mL and 51.9±7.1 mL per 100 g per minute, respectively. The gray/white matter (GM/WM) ratio of CBF was 3.0 for PET and 3.4 for pCASL. A paired *t*-test revealed differences in regional CBF between ASL and PET with higher ASL-CBF than PET-CBF values in cortical areas. Using an integrated, hybrid MR-PET a direct simultaneous comparison between ASL and [^15^O]water PET became possible for the first time so that temporal, physiologic, and functional variations were avoided. Regional and individual differences were found despite the overall similarity between ASL and PET, requiring further detailed investigations.

## Introduction

Positron emission tomography (PET) with ^15^O-labeled water is considered to be the gold standard for quantifying cerebral blood flow (CBF).^1^ However, because of the limited availability of PET in general, the need of an on-site cyclotron for the production of [^15^O]water, and the inherent radiation dose of the radiotracer, the MRI-based method of arterial spin labeling (ASL) has been recently considered as a viable alternative to determine CBF.^2, 3^

By using radiofrequency (RF)-labeled arterial blood as an intrinsic tracer, ASL can noninvasively measure CBF. However, a common problem with ASL is its low signal-to-noise ratio (SNR), which is the ratio of the average tissue signal intensity over standard deviation of the background noise. Low SNR is mainly because of the low fraction of blood within each voxel and T_1_ decay of the label.^4, 5^ To evaluate the reliability and reproducibility of ASL, several studies have compared ASL-MRI with [^15^O]water PET.^2, 3, 6, 7, 8^ Novel ASL techniques such as pseudo-continuous ASL (pCASL) deployed at 3T^9, 10, 11^ have substantially contributed to the attainment of high SNR (SNR=13.8) CBF data. Acceptable correlation has been shown between the absolute CBF as determined by pCASL and relative CBF from [^15^O]water PET.^2^ However, remaining differences between ASL and PET were reported.^8, 12^ Further, several studies have compared dynamic pulsed ASL without whole-brain coverage with whole-brain PET.^6, 7^ Some of the comparative studies investigated the relationship between ASL and PET in otherwise healthy subjects with a high risk of developing Alzheimer's disease and symptomatic carotid artery occlusion.^2, 6^ To avoid any effect on the quantitative CBF measurements owing to a possibly abnormal transit time in patients, a comparison between ASL and PET in healthy young subjects is preferable.

None of the above-mentioned CBF measurements compared ASL-MRI and [^15^O]water PET simultaneously so that temporal differences in functional and physiologic conditions cannot be excluded, especially considering that CBF is sensitive to a number of physiologic influences caused by caffeine intake, stress, state of arousal, or sleep deprivation.^13, 14, 15^

The most optimal way to avoid functional and physiologic variations in a multimodal study is to acquire data with different modalities simultaneously. Using an integrated, hybrid 3T MR-BrainPET scanner,^16, 17^ simultaneous measurements of whole brain and regional CBF with pCASL and [^15^O]water PET were performed, thereby ensuring that the same functional and physiologic variables equally affect both modalities. In this study, quantitative CBF parameters in a cohort of 10 healthy male subjects are reported.

## Materials and methods

### Subject Preparation

Ten healthy male volunteers with a mean age of 25±3 years (range 21 to 31 years) participated in the current study after providing written, informed consent; the study was approved by the ethics committee of the university hospital of RWTH Aachen University and federal authorities according to the Declaration of Helsinki ‘Ethical Principles for Medical Research Involving Human Subjects' and the German radiation protection law. Subjects were not allowed to consume any alcohol, caffeine, or nicotine for at least 12 hours before the scan. Before the examination, catheters were inserted into a radial artery and a contralateral antecubital vein. Bimodal CBF measurements consisted of simultaneous acquisition of pCASL and PET. These were performed during a baseline scan during which the subjects just had to watch a green square appearing occasionally with intervals varying randomly between 4.5 and 45 seconds on a black screen. There were three more scans with attention tasks during which the subjects had to react to the appearing green square in different manners. The whole study was performed twice after nights with and without sleep deprivation or vice versa in balanced, random order. This paper focuses on the methodological comparison of pCASL and [^15^O]water PET during the baseline scan after a night with sleep. The results of the entire study are still being analyzed and will be reported separately.

### 3T MR-BrainPET

All measurements were performed on a 3T MR-BrainPET developed by Siemens (Siemens Healthcare, Erlangen, Germany) as a prototype.^16, 17^ The BrainPET is operated as an insert within a slightly modified Siemens 3T MAGNETOM Trio MR scanner. The BrainPET insert is MR compatible and has high-resolution PET detectors with an axial field of view of 19.2 cm and an inner diameter of 32 cm. Each detector module has a 12 × 12 matrix of 2.5 × 2.5 × 20 mm^3^ individual lutetium oxyorthosilicate crystals coupled to a 3 × 3 array of avalanche photo diodes. Six detector modules are aligned within a copper-shielded cassette, and 32 such cassettes constitute the cylindrical PET detector that has an outer diameter of 60 cm enabling it to fit inside the bore of the MR scanner. Two dedicated MR head coils, consisting of an outer birdcage transmit coil and an inner 8-channel receive coil, were optimized with regard to minimal PET attenuation and are placed in the PET detector. PET images acquired with the MR-BrainPET have an excellent spatial resolution of 3 mm.^18^ Simultaneous acquisition of MRI and PET data sets can be carried out without any notable interference between the two modalities.^18^

### Arterial Spin Labeling

To perform ASL-MRI, a pCASL sequence was chosen because of its high SNR characteristics.^9, 10^ This sequence uses a 1.4-second train of RF and gradient pulses to invert the magnetization of blood water flowing through the labeling plane.^19^ In our experiments the position of the labeling plane was selected from a quick time-of-flight angiography to ensure optimal orientation of the carotid and vertebral arteries. Presaturation pulses were applied to the imaging region before labeling to reduce the static tissue signal and the physiologic noise. By using single-shot two-dimensional echo-planar imaging readouts, 100 measurements, i.e., 50 pairs of label-control volumes, were obtained. The sequence parameters used were as follows: flip angle/echo time/repetition time=90°/14/4,150 ms; matrix size: 64 × 64 × 26, partial Fourier=6/8; voxel size: 3.4 × 3.4 × 5 mm^3^; slice acquisition ordering: ascending, postlabeling delay=1 second, slice acquisition time=47 milliseconds, readout bandwidth=2,003 Hz; the total acquisition time was 7 minutes. CBF was then quantified using the ASL toolbox based on the Buxton model in MATLAB.^20, 21^ The timing difference of the postlabeling delay across the slices was corrected. Labeling efficiency was calibrated by an additional phase-contrast scan as reported by Aslan *et al.*^22^

### Positron Emission Tomography

**Figure 1:**
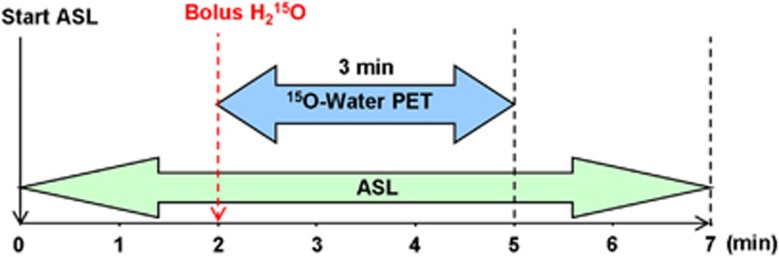
Timeline of the simultaneous measurement of [^15^O]water PET and ASL in a 3T hybrid MR-PET scanner. ASL takes 7 minutes and PET takes 3 minutes. After a bolus injection of 555 MBq [^15^O]water PET listmode data were recorded for 3 minutes. ASL, arterial spin labeling; MR, magnetic resonance; PET, positron emission tomography.

Immediately after the initiation of the pCASL sequence, a physician entered the scanner room through an RF lock system to administer the [^15^O]water injection. After 2 minutes of pCASL acquisition, 555 MBq [^15^O]water was intravenously injected as a short bolus and the PET listmode acquisition was started for 3 minutes (Figure 1). Approximately 60 seconds before the radiotracer injection, the measurement of arterial whole blood radioactivity was started and continued for ∼4 minutes using a continuous blood sampler (Swisstrace, Zürich, Switzerland). This sampler consists of an MR-compatible coincidence detector block with two lutetium–yttrium oxyorthosilicate crystals crystals and is shielded by tungsten. The outer dimensions of the detector block are 80 × 62 × 56 mm^3^. The light pulses of the crystals are transferred via two flexible light guides of 10 m length each outside the scanner room to photomultipliers in the readout device where the coincidence detection and the data storage take place. The withdrawal rate of the MR-compatible pump was 500 mL/hour. The length of the catheter tube with an inner diameter of 1 mm was ∼30 cm from the radial artery to the detector block dependent on the arm's length of the subject. The radiation dose caused by one injection of 555 MBq [^15^O]water is 0.63 mSv.

The listmode data were sorted into 30 frames of 4 seconds starting from the time of injection as well as into 1 frame of 60 seconds starting from the entry of the tracer into the brain. A fully three-dimensional ordinary Poisson ordered subset expectation maximization algorithm (2 subsets and 32 iterations)^23^ was used for image reconstruction. The reconstructed images were corrected for detector efficiency, random events, attenuation, scatter, dead time, and decay. For attenuation correction a template-based procedure was applied.^24^ This method uses an attenuation template derived from transmission scans of different subjects in an HR+ PET scanner and a corresponding MR template obtained from MP-RAGE (Magnetization Prepared Rapid Gradient Echo) images of the same subjects. The mean error obtained in cortical and subcortical regions with this method is 2.4%±3.7%.^24^ At the beginning of the entire imaging session an MP-RAGE image with a matrix of 192 × 256 × 256 voxels sized 1 mm^3^ was acquired. Using SPM the MP-RAGE template was nonlinearly registered to the individual MR template. The registration matrix was applied to the attenuation template, resulting in an individualized attenuation map, which was used together with attenuation maps of the MR head coils for the attenuation correction. The reconstructed image volume has 256 × 256 × 153 voxels with an isotropic voxel size of 1.25^3^ mm^3^. All images were postfiltered with a three-dimensional Gaussian kernel with 4 mm full-width at half-maximum (FWHM) so that the resulting image resolution was 5 mm at the center of the field of view based on the known intrinsic spatial resolution of the scanner and the smoothing filter FWHM. Quantitative CBF images (in units of mL per 100 g per minute) were derived using the autoradiographic PET image with a frame length of 60 seconds. We applied the one-tissue compartment model describing the cerebral kinetics of [^15^O]water and the autoradiographic method as suggested by Herscovitch *et al*^1^ for quantifying CBF. This approach is implemented in PMOD (Zürich, Switzerland), where a partition coefficient of *P*=0.9 was assumed. To obtain the input function required for the CBF calculation, the measured blood radioactivity was corrected first for radioactive decay with respect to the start of PET acquisition and subsequently for delay and dispersion. For the latter correction a one-compartment model was used with the measured decay-corrected blood curve and the time–activity curve of the whole brain as input data. The dispersion time constant was 8.7±1.7 seconds and the delay was 12.5±3.4 seconds.

### Additional Issues Related to the Simultaneous MR/PET Study Design

There are a number of challenges that have to be considered in a simultaneous MR/PET study in general and in that of CBF specifically. There is no possibility to measure the tissue attenuation of the PET data such as in PET/CT. Therefore, a dedicated procedure for attenuation correction as mentioned above must be applied. Furthermore, the additional radiation absorption by the head coils and ear phones must be taken into account. In MR/PET specific head coils are designed to cause less radiation absorption compared with conventional ones. Nevertheless, the still existing absorption must be considered by the attenuation correction. Whereas this correction can be achieved quite easily owing to the known fixed position of the coils, the radiation absorption caused by ear phones commonly used in MRI cannot be corrected straightforwardly, since their position is not exactly known. Therefore, we replaced the ear phones by ear plugs, which have a central tube, and connected them to the pneumatic cables coming from the operator's room. The absorption by the ear plugs could be neglected.

A further challenge is the timely preparation of the [^15^O]water with its fast radioactive decay of 2 minutes and the synchronization of the injection with the ASL measurement. Here one must ensure that the desired amount of ∼555 MBq [^15^O]water is ready to be injected 2 minutes after the start of the ASL sequence.

### Statistical Analysis

All images were postprocessed using SPM8 software (Welcome Trust Centre for Neuroimaging, London, UK). The ASL-CBF images were smoothed to a resolution of 5 mm using a 4-mm FWHM Gaussian kernel. Individual MP-RAGE data set from each subject was normalized into the Montreal Neurological Institute (MNI) space, and the transformation was applied to the PET- and ASL-CBF images. The PET- and ASL-CBF images were then resliced and registered to the MP-RAGE data set. Whole-brain CBF values were calculated within a volume of interest (VOI) comprising the entire brain of the averaged (*n*=10) ASL-CBF image. The mean CBF values for GM were obtained from VOIs defined by contour thresholding the averaged ASL-CBF image at three adjacent slices at the level of the basal ganglia. To obtain CBF values for WM, four small regions of interest were placed in the WM regions of each of the three slices. In this way, spillover from GM areas could be avoided. In addition, to examine the regional CBF, 10 different VOIs from the AAL (automatic anatomical labeling^25^) template offered in the VOI tool of PMOD were defined over eight cortical areas, the thalamus and the cerebellum. To examine possible correlations between PET- and ASL-CBF for the different VOIs across all subjects, the Pearson and Spearman correlation coefficients were used and tested for significance. Furthermore, voxel-by-voxel differences between CBF images of ASL and PET across the 10 subjects were compared using a paired *t*-test with SPM. The option of analysis of covariance was chosen and grand mean scaling was not chosen.

## Results

**Figure 2:**
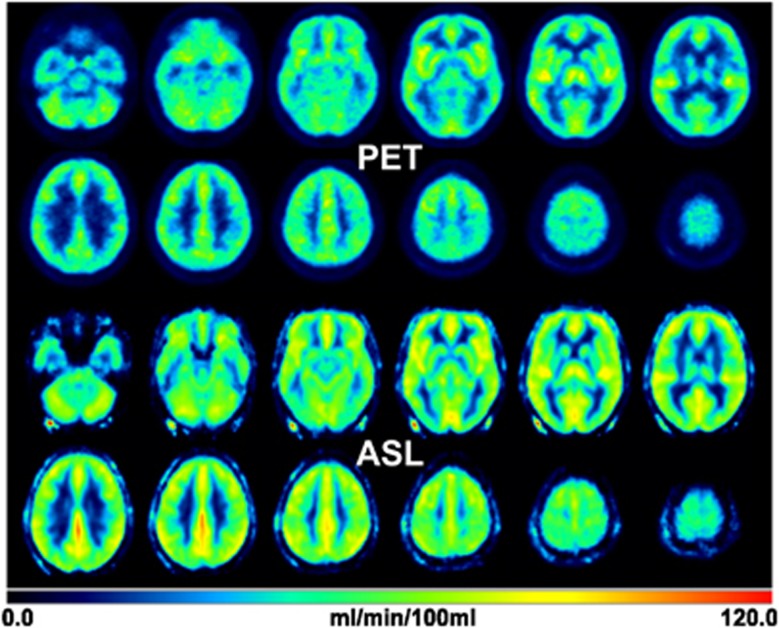
Averaged PET and ASL-CBF images (*n*=10) after normalization into the MNI space. The CBF values from both methods show an agreement in the value range of 0 to 120 mL per 100 g per minute. The averaged whole-brain CBF from PET is 43.3±6.1 mL and 51.9±7.1 mL per 100 g per minute from ASL. ASL, arterial spin labeling; CBF, cerebral blood flow; MNI, Montreal Neurological Institute; PET, positron emission tomography.

A visual comparison of the averaged (*n*=10) PET-CBF and ASL-CBF maps in MNI space reveals a good overall agreement (Figure 2). Both the PET and ASL data fit well within the color scale ranging from 0 to 120 mL per 100 g per minute without lower and upper cutoffs. Averaged whole-brain CBF of the 10 subjects measured at baseline condition after sleep was 43.3±6.1 mL per 100 g per minute for PET and 51.9±7.1 mL per 100 g per minute for ASL. Compared with PET, the averaged CBF in GM measured by ASL is higher (67.3±8.2 versus 51.8±7.7 mL per 100 g per minute) and the averaged CBF in WM is similar (19.5±5.8 versus 17.4±3.1 mL per 100 g per minute), which results in a GM/WM ratio of 3.4 and 3.0 for ASL and PET, respectively. A closer look at the WM results revealed a variation within the WM VOIs for ASL-CBF, which was twice the variation for PET-CBF. The mean (*n*=10) coefficient of variation in the WM VOIs was 56% for ASL-CBF and 29% for PET-CBF. In comparison, the corresponding results for GM were 23% and 32%.

**Figure 3:**
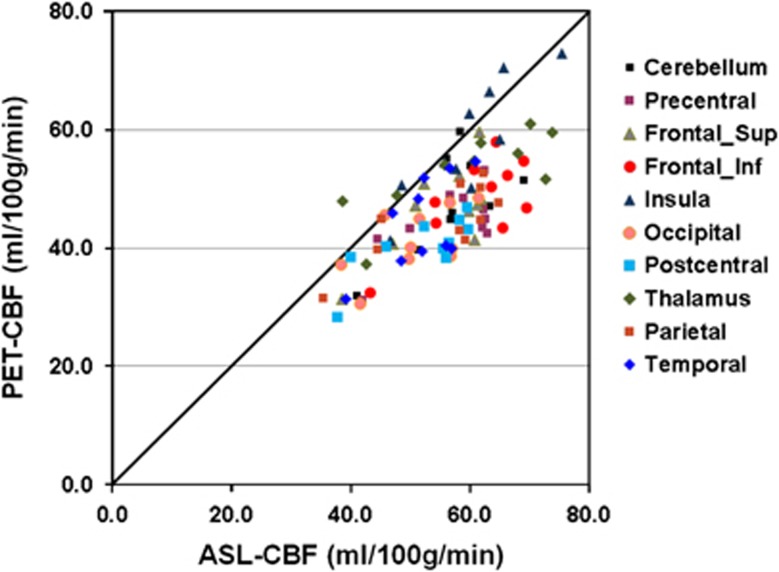
Regional comparison of PET-CBF and ASL-CBF measured across the 10 subjects in eight cortical volumes of interest, the thalamus and the cerebellum. ASL, arterial spin labeling; CBF, cerebral blood flow; PET, positron emission tomography.

Figure 3 presents scatter plots of CBF data of eight cortical VOIs, the thalamus and the cerebellum. When testing the correlation between PET-CBF and ASL-CBF with the Pearson correlation, 9 of 10 VOIs showed a significant (*P*<0.05) result, whereas the Spearman correlation yielded a significant correlation only for four VOIs (Table 1). The Pearson and Spearman correlation coefficients between the PET-CBF and ASL-CBF across all 10 VOIs were both significant (*P*<0.005) with values of 0.81 and 0.88, respectively.

**Figure 4:**
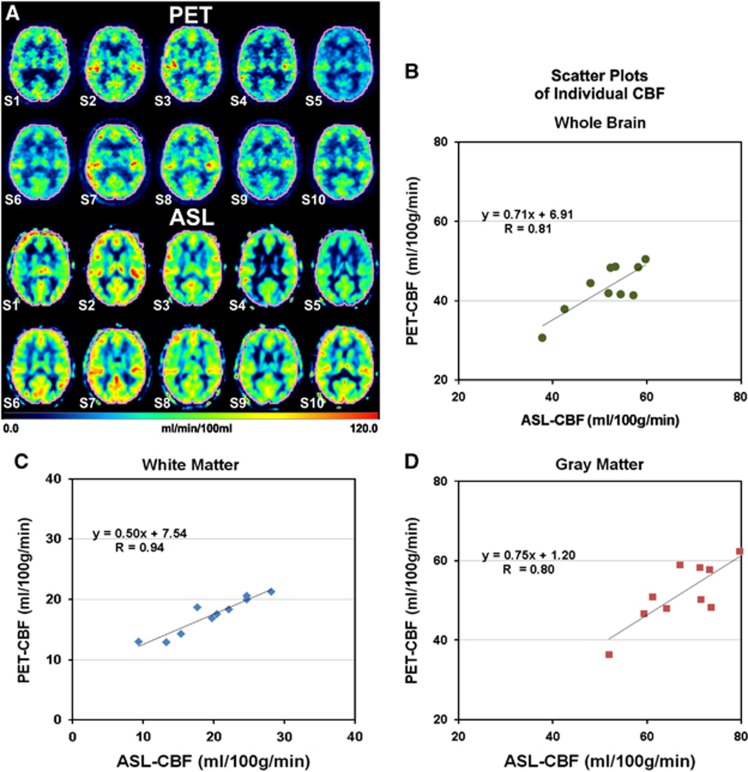
Comparative images of PET-CBF and ASL-CBF at the level of the basal ganglia in the individual subjects S1 to S10 (**A**). All images are normalized to the MNI space. A VOI (in pink) comprising the entire brain was defined for the calculation of the whole-brain CBF. Scatter plots of VOI averages of CBF in the whole brain (**B**), white matter (**C**), and gray matter (**D**) indicate positive correlations between PET-CBF and ASL-CBF. *R* is the Pearson correlation coefficient. ASL, arterial spin labeling; CBF, cerebral blood flow; MNI, Montreal Neurological Institute; PET, positron emission tomography; VOI, volume of interest.

**Figure 5:**
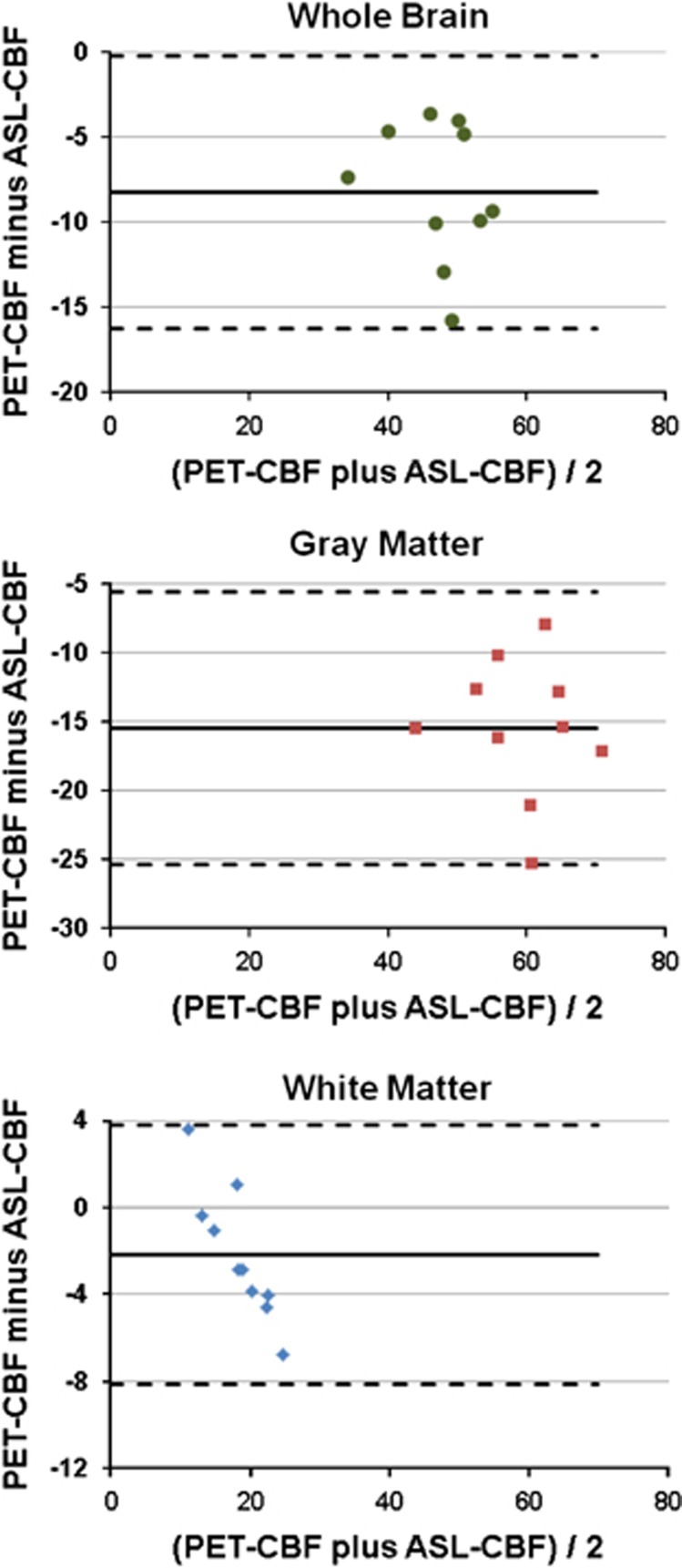
Bland–Altman plots of differences between PET-CBF and ASL-CBF volume of interest averages of CBF in the whole brain, gray matter, and white matter of the 10 subjects. The average difference is indicated by the solid line, whereas the dashed lines represent the ±2 standard deviations. ASL, arterial spin labeling; CBF, cerebral blood flow; PET, positron emission tomography.

The individual CBF data of all 10 subjects, S1 to S10, measured in the VOIs of whole brain, GM, and WM are presented in Figure 4. A comparison of individual CBF images at the level of the basal ganglia obtained with ASL and PET in each of the 10 subjects, S1 to S10, is provided in Figure 4A. There is good overall agreement of the individual images acquired with the two modalities; that is, low ASL-CBF images match with low PET-CBF images and vice versa. However, a closer examination reveals local differences between the two methods. The individual, whole-brain CBF data obtained with ASL and [^15^O]water PET, respectively, in the 10 subjects are compared in Figure 4B. The scatter plot shows a high correlation between ASL and PET with a Pearson correlation coefficient of 0.81 (*P*<0.005) and Spearman correlation coefficient of 0.65 (*P*<0.05). The corresponding comparisons of GM and WM CBF data are shown in Figures 4D and C, respectively. Here again positive correlations were observed between ASL and PET across the 10 subjects with Pearson correlation coefficients of 0.80 (*P*<0.01) and 0.94 (*P*<0.001), respectively, and Spearman correlation coefficients of 0.64 (*P*<0.05) and 0.90 (*P*<0.001), respectively. Furthermore, Bland–Altman plots of the CBF data resulting from ASL and [^15^O]water PET show small ranges of differences between the two modalities (Figure 5). For whole brain, GM, and WM the difference in CBF between ASL and PET is within the two standard deviation lines (0.95 confidence interval).

**Figure 6:**
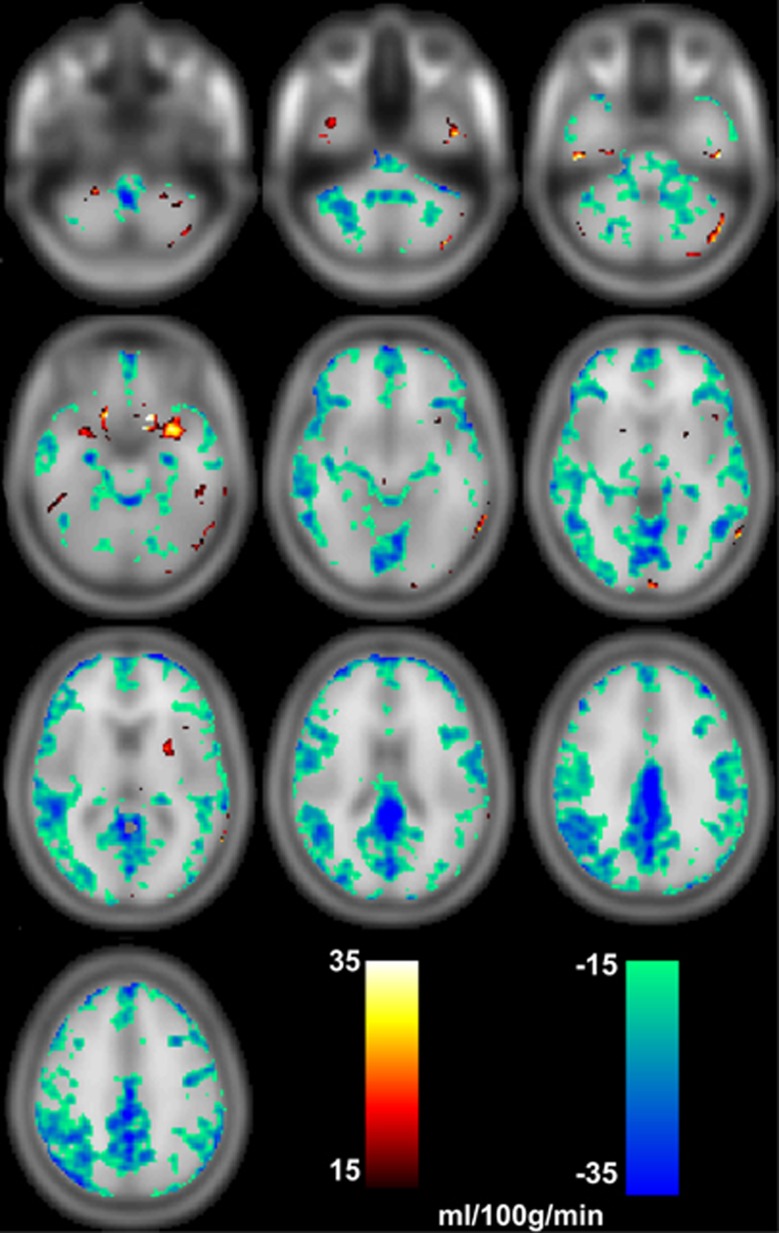
Images PET-CBF minus ASL-CBF showing differences (*P*<0.01 uncorr.) in the ranges of 15 to 35 mL per 100 g per minute and −15 to −35 mL per 100 g per minute. Especially in cortical regions CBF measured by ASL is higher than measured by PET. ASL, arterial spin labeling; CBF, cerebral blood flow; PET, positron emission tomography.

This latter finding is supported by an SPM-based paired *t*-test of voxel-by-voxel CBF. Across the 10 subjects, absolute differences ranging from 15 to 35 mL per 100 g per minute (*P*<0.01 uncorr.) were found predominantly in GM (Figure 6).

## Discussion

The CBF measured in this study by pCASL with an average of 51.9±7.1 mL per 100 g per minute is generally higher than the CBF measured by [^15^O]water PET with an average of 43.3±6.1 mL per 100 g per minute. Lassen considered 50 mL per 100 g per minute as the normal whole-brain CBF in young adults.^26^ The results obtained here also do not differ significantly from a report by Herscovitch *et al*,^27^ who determined the whole-brain CBF value of 53.1 mL and 44.4 mL per 100 g per minute using [^11^C]butanol and [^15^O]water, respectively, as PET-CBF tracers. A previous PET study from our group found a whole-brain CBF of 49.1±8.0 mL per 100 g per minute using [^15^O]butanol in 27 healthy young subjects.^28^ Whole-brain CBF values ranging from 51 to 56 mL per 100 g per minute have also been previously reported for CASL.^29, 30, 31^ One may assume pCASL is closer to PET-CBF measured with radiolabeled butanol than with [^15^O]water.

The 18% higher whole-brain CBF resulting with pCASL is primarily based on the 29% higher blood flow measured in GM. In WM, ASL-CBF was 12% higher than PET-CBF. The greater difference between ASL-CBF and PET-CBF in GM compared with the difference in WM is also expressed by the greater gray/white ratio of 3.4 for ASL compared with 3.0 for PET.

Despite these quantitative differences, a comparison between pooled CBF images as presented in Figure 2 shows a great similarity between both methods. Figure 4 also shows the similarity between PET-CBF and ASL-CBF. In those subjects (e.g., S2 and S7) in whom the PET images indicate increased CBF the ASL images do it as well and vice versa (e.g., S4 and S5). This observation is supported by the correlations between PET-CBF and ASL-CBF in whole brain, GM, and WM (Figures 4B–D), as well as in eight cortical VOIs, the thalamus and the cerebellum (Figure 3 and Table 1).

Although the mean CBF in WM is similar in ASL and PET with a relative difference of 12%, there is a much greater variation at the voxel level in WM VOIs for ASL-CBF. There are voxels with values near zero in WM for ASL-CBF. One reason for such obviously underestimated CBF values in WM by ASL is the short postlabeling delay of 1 second. In the ASL analysis it was assumed that the tagged blood has arrived in each readout voxel after the delay. If the arterial transit time is longer than the postlabeling delay (e.g., in WM), CBF will be underestimated. Experimental studies^32^ have shown that a postlabeling delay between the labeling and readout in pCASL should be longer than 1.5 seconds for an improved measurement of WM CBF.^29^ Variations in the bolus arrival time can occur across brain regions and in areas with disease, which represents a problem for selecting an optimized postlabeling delay. Although a long delay is favorable to obtain more accurate values for WM CBF, it leads to a lower SNR owing to the magnetization decay. A dynamic ASL study with varying postlabeling delay could reduce the signal sensitivity to bolus arrival time, and also needs a longer measurement time with fewer slices or less averages than used in our study.^30^ One factor that can affect the quantification of CBF in ASL is the labeling efficiency. When a low labeling efficiency (in our case 0.86) is determined by the whole-brain perfusion using phase contrast,^22^ a high CBF measured by ASL may result.

Although PET and ASL images were smoothed to obtain a similar image resolution of ∼5 mm there might have been an additional factor decreasing the resulting GM CBF when measured by [^15^O]water PET. The BrainPET detector has a decreasing resolution in radial direction from 3 mm in the center to 4.5 mm at a radius of 7.5 cm because of its small ring diameter and the associated depth of interaction effect.^17, 18^ Thus, the partial volume effect is expected to be slightly greater in cortical regions compared with central ones.

In contrast to PET, higher CBF values in ASL were found in several regions around the big vessels (Figure 6). These regional differences in the mid-frontal region and posterior cingulate were also noticed in a previous study.^30^ The ASL signal can be easily contaminated by the presence of tagged blood in arterial vessels that is destined to perfuse tissue in more distal regions. This intravascular overestimation will be particularly significant near major arteries such as the anterior, middle, and posterior cerebral arteries,^31^ and can also be observed in Figure 2 at the location of the superficial cranial arteries around the brain. To suppress the intravascular flow signal, crushing gradients can be added,^31, 33^ or a multidelay approach can be combined with signal modeling to remove the macrovascular signal in postprocessing.^31^

Further discrepancies in regional CBF between ASL and PET due to susceptibility effects at air–tissue interfaces and image distortions from the echo-planar imaging readout in ASL can be observed at the location of the inferior temporal lobe and the paranasal sinuses (see the Supplementary Figure). Such distortions might be minimized by optimizing the ASL readout scheme and shimming.

A number of previous studies performed sequential measurements with ASL and [^15^O]water PET.^2, 3, 6, 7, 8^ Two of these studies did not report quantitative CBF data for the PET measurements and scanned patients with Alzheimer's disease^2^ or healthy subjects under different glycemic conditions.^3^ Thus, it is not sensible to compare their results with those presented here. Bokkers *et al*^6^ examined patients with symptomatic carotid artery occlusion and applied ‘image acquisition at multiple inversion times to compensate for spatial heterogeneities in transit times caused by collateral blood flow in patients with severe stenosis'. Interestingly, in anterior GM they found CBF values higher by 25% with ASL than with PET, but in posterior GM the CBF values with ASL were approximately twice those with PET. As indicated in Figure 3 such differences were not present in our study. Henriksen *et al*^7^ compared the intersubject variability of ASL and two other MRI CBF methods with [^15^O]water PET. They found whole-brain CBF of 37.0 mL per 100 g per minute with ASL and 41.9 mL per 100 g per minute with PET, a nearly identical WM CBF (21.7 mL versus 22.6 mL per 100 g per minute), and greater GM PET-CBF of 58.6 mL per 100 g per minute compared with an ASL-CBF of 50.2 mL per 100 g per minute. The latter finding is just the opposite of the results reported here. In that study, PET imaging took 7 minutes for each scan and Alpert's one compartment model was applied for CBF quantitation.^34^ Qiu *et al*^8^ applied CBF mapping with pulsed ASL. Using PET as the ‘gold standard' they showed how the arterial transit time affects CBF quantitation with ASL.

Use of an integrated, hybrid MR-PET scanner capable of simultaneous acquisition of both modalities is the main feature of this study. CBF is believed to be tightly coupled to neuronal activity and is affected by various factors.^35^ A previous study with [^15^O]water PET reported an 8% variation in WM and a 10% variation in GM in 48 hours.^36^ An ASL study has reported that the variation within a 1-week interval was ∼14% for the whole brain and regional CBF, and the difference was attributed more to variation in physiology over time and less to measurement error.^37^ Thus, such variations are supposed to be relevant in sequential CBF measurements with ASL and [^15^O]water. With an integrated, hybrid system patients can be simultaneously scanned in a ‘one-stop shop' approach and in the same underlying physiologic condition for both [^15^O]water PET and ASL measurements. Moreover, once validated, ASL could be used to provide CBF measures and PET could be used with another tracer of choice to enable different aspects of the brain to be studied simultaneously.

In order to realize true simultaneous CBF measurements with [^15^O]water PET and ASL-MRI, a specific construction of the MR room and additional instrumentation was necessary. The entrance from the operator room to the MR room has a double door, which functions as an RF lock so that the MR room can be accessed at any time without breaking the RF seal. Thus, [^15^O]water can be injected during the time the ASL sequence is running. For continuous blood sampling an MR-compatible detector frontend was used, as well as an MR-compatible pump, which could be operated while the ASL sequence was going on.

When comparing ASL and [^15^O]water PET, one may argue that the MR-based method is superior in spatial and temporal resolution. Generally, MRI has a better spatial resolution than PET, especially if anatomic imaging with T_1_-weighted sequences, such as MP-RAGE, is considered. The optimal image resolution (expressed as FWHM) achievable with PET at the center of the field of view was ∼5 mm, because the reconstructed images had to be postfiltered with a three-dimensional Gaussian kernel of 4 mm to reduce image noise. The parameters describing image resolution in PET and ASL are not directly comparable. The FWHM of a point spread function represents the resolution in PET, whereas in MRI the voxel size is regarded as such a parameter. In our case the voxel size of ASL is 3.4 × 3.4 × 5 mm^3^, which is considerably bigger than that of standard anatomic MR images. The argument for the better temporal resolution of MRI may be derived from the fact that an event-related neuroactivation study is possible with functional MRI, but not with PET. Furthermore, there are PET studies with a recording time of many minutes or even some hours. In the case of the present [^15^O]water PET study the acquisition time was only 3 minutes, of which 1 minute was necessary for obtaining a parametric quantitative CBF image of the entire brain. Deriving ASL-CBF data with whole-brain coverage and a voxel size of 3.4 × 3.4 × 5 mm^3^ required 7 minutes. A second ASL scan can follow the first one directly. In contrast, PET can be started only 10 minutes after the preceding injection in order to allow ^15^O to decay sufficiently. ASL scans with smaller voxels might be obtained if the measurement time is prolonged or fewer planes are recorded. However, with fewer planes there is no whole-brain coverage that is delivered by PET *per se*. In summary, the spatial and temporal resolutions of [^15^O]water PET and ASL are similar, if the entire brain is to be imaged as in the present study.

Future improvements in the ASL technique, such as multiple postlabeling delay, can be expected, resulting in clearer spatial and temporal advantages of ASL compared with the sequence used here. Furthermore, the fact that ASL-based methods use an intrinsic, non-radioactive tracer remains a potent advantage especially in longitudinal studies or in studies with repeated measurements. Simultaneous PET-MRI is the modality of choice to validate the further development of CBF measurements with ASL.

## Conclusion

The feasibility of simultaneous measurements of [^15^O]water PET and ASL-MRI has been shown using a 3T hybrid MR-PET brain scanner. For the first time, a direct comparison under identical physiologic conditions of the two modalities has been performed in humans. The CBF images obtained in this study by both [^15^O]water PET and ASL-MRI were similar in qualitative respect and were quantitatively higher for ASL. The remaining differences may require further investigation.

## Figures and Tables

**Table 1:** Comparison of regional ASL-CBF and PET-CBF

	*ASL*		*PET*		*Pearson*	*Spearman*
*VOI*	*Mean*	*s.d.*	*Mean*	*s.d.*	*Corr. coef.*	*Corr. coef.*
Precentral	56.4	8.0	44.4	5.9	0.70*	0.41
Frontal_Sup	55.3	7.8	46.6	7.6	0.70*	0.58
Frontal_Inf	61.2	8.3	48.2	7.3	0.72*	0.37
Insula	60.0	8.4	58.1	10.0	0.87*	0.82*
Occipital	50.9	7.4	41.1	5.5	0.61	0.60
Postcentral	52.2	8.1	40.5	5.1	0.77*	0.70*
Thalamus	59.3	12.7	52.8	7.0	0.78*	0.77*
Parietal	55.3	9.8	44.6	6.3	0.78*	0.71*
Temporal	52.1	6.2	44.3	7.7	0.65*	0.61
Cerebellum	57.1	7.3	47.8	8.0	0.70*	0.54
10 VOIs Pearson corr. coef.	0.81**					
10 VOIs Spearman corr. coef.	0.88**					

ASL, arterial spin labeling; CBF, cerebral blood flow; coef., coefficient; corr., correlation; PET, positron emission tomography; s.d., standard deviation; VOI, volume of interest; significances: *P<0.05, **P<0.005.
