# Supplementary figures and images for: Comparison of cerebral blood flow acquired by simultaneous [15O]water positron emission tomography and arterial spin labeling magnetic resonance imaging
Source: J Cereb Blood Flow Metab. 2014 May 21;34(8):1373–80. doi: 10.1038/jcbfm.2014.92 (PMC4126098; doi:10.1038/jcbfm.2014.92)

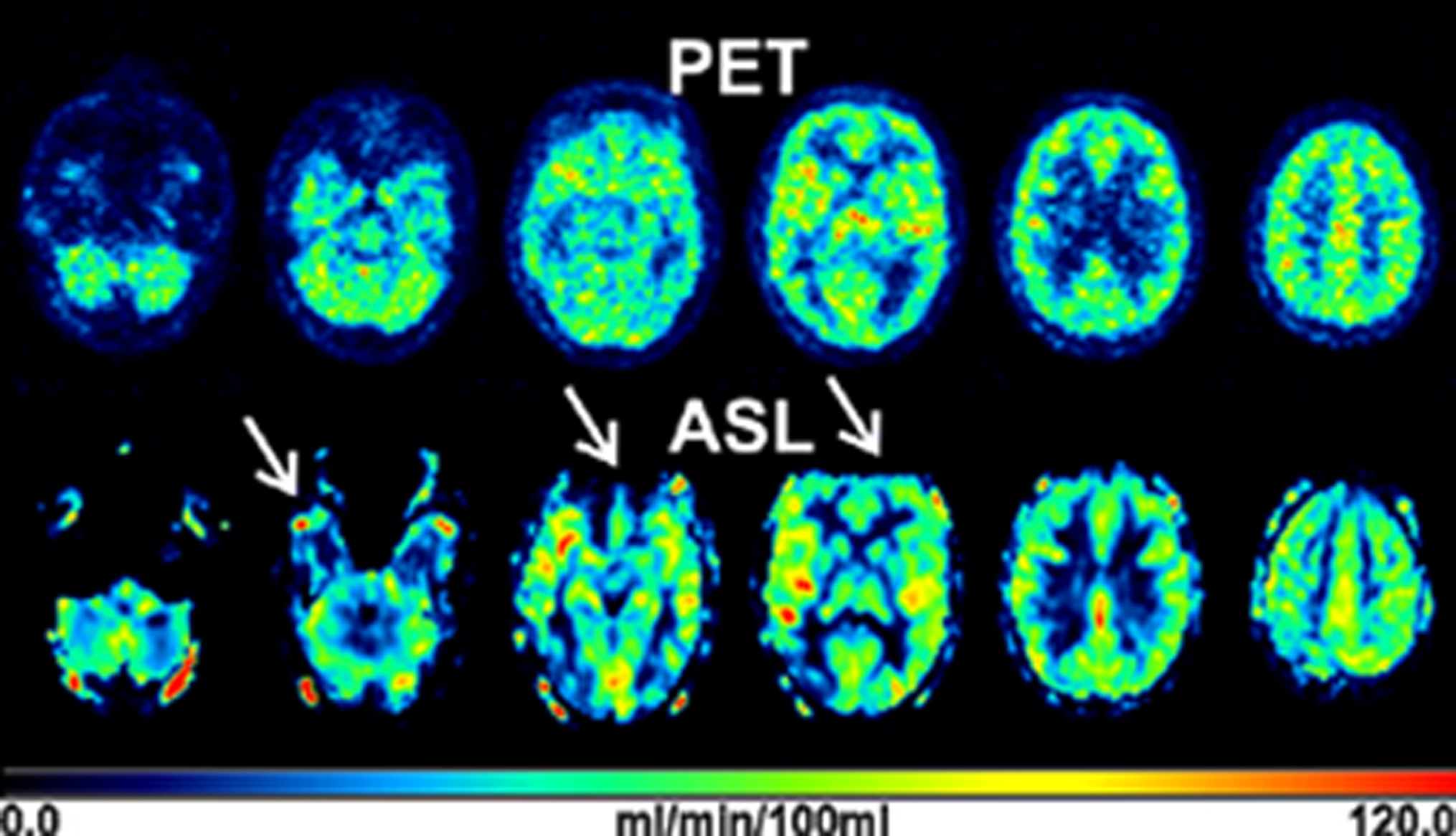

Supplement: Supplementary Figure [file jcbfm201492x1.tif]
