# Supplementary material for: Comparison of cerebral blood flow acquired by simultaneous [15O]water positron emission tomography and arterial spin labeling magnetic resonance imaging
Source: J Cereb Blood Flow Metab. 2014 May 21;34(8):1373–80. doi: 10.1038/jcbfm.2014.92 (PMC4126098; doi:10.1038/jcbfm.2014.92)
Supplement: Supplementary Figure Legend [file jcbfm201492x2.doc]

**Supplementary figure legend**

Supplementary figure: Discrepancies between ASL and PET due to image distortions shown for a representative subject. Signal voids can be found at locations with strong field changes such as inferior temporal lobe, sinusoid and frontal lobe (arrows).
